# Effect measure modification of the association between short-term exposures to PM_2.5_ and hospitalizations by long-term PM_2.5_ exposure among a cohort of people with Chronic Obstructive Pulmonary Disease (COPD) in North Carolina, 2002–2015

**DOI:** 10.1186/s12940-023-00999-4

**Published:** 2023-06-29

**Authors:** Kristen N. Cowan, Lauren H. Wyatt, Thomas J. Luben, Jason D. Sacks, Cavin Ward-Caviness, Kristen M. Rappazzo

**Affiliations:** 1 Department of Epidemiology, GillingsSchool of Global Public Health, University of North Carolina, Chapel Hill, USA; 2 Oak Ridge Institute for Science and Education (ORISE) at US EPA, Oak Ridge, USA; 3 U.S. Environmental Protection Agency, Office of Research and Development, 109 T.W. Alexander Dr, MD 58C, Research Triangle Park, Durham, NC 27711 USA

**Keywords:** Air Pollution, Environmental Epidemiology, Chronic Obstructive Pulmonary Disease (COPD)

## Abstract

**Background:**

Approximately nine million adults in the United States are living with chronic obstructive pulmonary disease (COPD), and positive associations between short-term air pollution exposure and increased risk of COPD hospitalizations in older adults are consistently reported. We examined the association between short-term PM_2.5_ exposure and hospitalizations and assessed if there is modification by long-term exposure in a cohort of individuals with COPD.

**Methods:**

In a time-referent case-crossover design, we used a cohort of randomly selected individuals with electronic health records from the University of North Carolina Healthcare System, restricted to patients with a medical encounter coded with a COPD diagnosis from 2004–2016 (n = 520), and estimated ambient PM_2.5_ concentrations from an ensemble model. Odds ratios and 95% confidence intervals (OR (95%CI)) were estimated with conditional logistic regression for respiratory-related, cardiovascular (CVD), and all-cause hospitalizations. Exposures examined were 0–2 and 0–3 day lags of PM_2.5_ concentration, adjusting for daily census-tract temperature and humidity, and models were stratified by long-term (annual average) PM_2.5_ concentration at the median value.

**Results:**

We observed generally null or low-magnitude negative associations with short-term PM_2.5_ exposure and respiratory-related (OR per 5 µg/m^3^ increase in 3-day lag PM_2.5_: 0.971 (0.885, 1.066)), CVD (2-day lag: 0.976 (0.900, 1.058) and all-cause (3 day lag: 1.003 (0.927, 1.086)) hospitalizations. Associations between short-term PM_2.5_ exposure and hospitalizations were higher among patients residing in areas with higher levels of annual PM_2.5_ concentrations (OR per 5 µg/m^3^ in 3-day lag PM_2.5_ for all-cause hospitalizations: 1.066 (0.958, 1.185)) than those in areas with lower annual PM_2.5_ concentrations (OR per 5 µg/m^3^ in 3-day lag PM_2.5_ for all-cause hospitalizations: 0.914 (0.804, 1.039)).

**Concluisons:**

Differences in associations demonstrate that people in areas with higher annual PM_2.5_ exposure may be associated with higher risk of hospitalization during short-term increases in PM_2.5_ exposure.

**Supplementary Information:**

The online version contains supplementary material available at 10.1186/s12940-023-00999-4.

## Background

Chronic Obstructive Pulmonary Disease (COPD), a group of diseases related to air flow blockage and breathing problems including emphysema and chronic bronchitis [1], is a common disease among older adults. In the United States, 6.4% of people report that they have been diagnosed with COPD [2]. People aged 65 years and older are at higher risk of developing COPD and in 2018 COPD was the fourth leading cause of death [3]. Smoking, occupational exposures, respiratory infections and air pollution are all factors associated with the development of COPD, and a number of studies have identified smoking and ambient air pollution as the largest drivers of COPD incidence globally [4–6]. Not only are these factors associated with the development of COPD, but for those with COPD, smoking and air pollution exposure are known to exacerbate respiratory symptoms [7–10].

Both short-(i.e., days up to a few weeks) and long-term (i.e., months, annual, multi-year) exposures to air pollution, specifically fine particulate matter (PM_2.5_, particles with an aerodynamic diameter generally ≤ 2.5 µm) have been associated with respiratory and cardiovascular effects, as well as mortality [11–15]. Many studies have shown that short-term PM_2.5_exposure is associated with respiratory effects ranging from symptoms to more severe effects, such as respiratory-related hospitalizations [16, 17]. Some studies provide evidence that patients with COPD are more vulnerable to these effects including COPD exacerbations [18–20]. Short-term PM_2.5_ exposure is also associated with increases in hospital visits for cardiovascular disease [21–23] as well as all-cause hospitalizations [24–27]. Studies examining long-term exposure to air pollution have reported associations between PM_2.5_and mortality, with some studies providing initial evidence of increased risk of hospitalization [28]. Additionally, the burden of ambient air pollution is not equally distributed, with communities of color and low-income communities tending to have the poorest air quality [29–31]. Despite improvements in air quality, exposure to ambient air pollution continues to be a public health concern [32–37].

There is, however, limited understanding of interactions between long- and short-term PM_2.5_ concentration exposures. There is potential that continuous exposure to higher concentrations of PM_2.5_ might cause the body to acclimate and thus those who live in areas of higher annual PM_2.5_ concentrations may experience a lower risk of hospitalization when there are short-term spikes in PM_2.5_ concentrations. Alternatively, it may be that those who are exposed to higher long-term concentrations accumulate insults to their physical health over time and may experience higher risks of hospitalization when there are short-term spikes in PM_2.5_ concentrations. However, there is not a substantial body of research addressing this topic, or the complexities of examining air pollution exposures in at-risk populations, such as COPD patients.

The goals of this study were to (1) estimate the association between short-term PM_2.5_ exposure and hospitalizations among patients with a medical encounter coded with COPD in North Carolina and (2) to examine if this association differs for those people living in areas with higher annual PM_2.5_ concentrations compared to those living in areas with lower annual concentrations of PM_2.5_. Outcomes examined include all-cause, respiratory-related and cardiovascular-related hospitalizations.

## Methods

### Study population

Data for this study was obtained from a randomly selected cohort of individuals accessing the University of North Carolina Healthcare System from April 2004-July 2016, identified using electronic health records (EHRs). Individuals were excluded from this cohort if they did not have an address that could be geocoded. Details about the data, cohort, and geocoding have been published previously [38]. From the overall cohort, a sub-cohort of patients with COPD was selected by identifying those who had any visit during the timeframe of interest (2004–2016) with an ICD-9 or ICD-10 code for COPD (Table 1) and had one or more hospitalizations at least thirty days after their first COPD-related visit (*n* = 520), which provides a similar “entrance” point to our analysis (initial COPD visit) for all included individuals.

**Table 1:** ICD-9 and ICD-10 Codes used for classification

Condition	ICD-9 Codes	ICD-10 Codes
Chronic Obstructive Pulmonary Disease (COPD)	491.X, 492.X, 496.X	J41.X, J42.X, J43.X, J44.X
Respiratory-related Conditions	460.X, 461.X, 462.X, 463.X, 464.X, 465.X, 466.X, 470.X, 471.X, 472.X, 473.X, 474.X, 475.X, 476.X, 477.X, 478.X, 480.X, 481.X, 482.X, 483.X, 484.X, 485.X, 486.X, 487.X, 488.X, 490.X, 491.X, 492.X, 493.X, 494.X, 495.X, 496.X, 500.X, 501.X, 502.X, 503.X, 504.X, 505.X, 506.X, 507.X, 508.X, 510.X, 511.X, 512.X, 513.X, 514.X, 515.X, 516.X, 517.X, 518.X, 519.X	J0.X, J1.X, J2.X, J3.X, J4.X, J5.X, J6.X, J7.X, J8.X, J9.X
Cardiovascular-related conditions	401.X, 402.X, 403.X, 404.X, 405.X, 410.X, 411.X, 412.X, 413.X, 414.X, 415.X, 416.X, 417.X, 420.X, 421.X, 422.X, 423.X, 424.X, 425.X, 426.X, 427.X, 428.X, 429.X	I1.X, I2.X, I3.X, I4.X, I5.X

### Health outcomes

The outcomes of interest for this analysis were cardiovascular-related hospitalizations, respiratory-related hospitalizations, and all-cause hospitalizations, extracted from EHRs. To be identified as an outcome included in the case-crossover cohort, the visit had to occur at least 30 days after the patient’s first COPD hospitalization and match an ICD-9 or ICD-10 code primary diagnosis for the conditions described in Table 1. Any hospitalizations that occurred within the admission and discharge window of another hospitalization were deemed hospital transfers and were removed from the dataset.

### Exposure data and covariates

Daily (24-h average) PM_2.5_ (μg/m^3^) concentrations were estimated using a previously described ensemble model that includes satellite data, meteorological data, chemical transport model data, elevation, and land-use, along with air pollution monitoring data [39]. Daily concentration estimates for each 1km^2^grid centroid from the ensemble model were averaged across the 2010 census tract in which they fell. The lags examined varied by outcome with a 0–2 day lag for cardiovascular outcomes, 0–3 day lag for respiratory outcomes, and both 0–2 and 0–3 day lags for all-cause hospitalizations. A shorter time period from exposure to outcome has been documented for cardiovascular effects than respiratory effects which led to different lag-periods being used for different outcomes [40, 41]. Long-term exposure was represented by annual average PM_2.5_ concentration, using the daily PM_2.5_concentrations averaged across the 364 days prior to first COPD hospitalization. Data on daily temperature and relative humidity for 2010 census tracts were acquired from the North American Land Data Assimilation System Phase 2 (NLDAS) model, after conversion using a multistage geo-imputation approach [42]. Air pollution, temperature and humidity data were linked to patient EHR based on the 2010 census tract into which their residential address falls.

### Case-crossover design and statistical analysis

We used a case-crossover design, in which individuals serve as their own controls, to estimate associations between short-term PM_2.5_exposure and hospitalization [43–46]. For the analysis, control periods were selected for each outcome event (hospitalization) using a time-stratified referent selection approach; each event could have up to four control periods, selected as a non-event on the same day of the week within the same calendar month and year. For example, if someone had a hospital visit on Wednesday, March 18, 2015, they would be assigned control dates of Wednesday, March 4, 2015, Wednesday, March 11, 2015, and Wednesday, March 25, 2015. Once control dates were created for all cases, daily average PM_2.5_concentration, temperature and relative humidity were assigned to each case and control date with 0–2 day lag for cardiovascular outcomes, 0–3 day lag for respiratory outcomes, and both 0–2 and 0–3 day lags for all-cause hospitalizations [40, 41].Because case-crossover studies automatically control for time-invariant factors by design, only time-varying confounders (i.e., daily census-tract temperature and humidity) were included in adjusted models.

Conditional logistic regression was used to estimate the odds ratio and the 95% confidence interval (OR (95%CI)) per a 5 μg/m^3^ increase in PM_2.5_ exposure and hospitalization; a 5 μg/m^3^ increase was used as this is approximately the IQR for PM_2.5_ exposure in our cohort. Because individuals could experience multiple hospitalization events over the study period, a term stratifying by patient ID was also included in the models to allow for multiple hospitalizations from the same person to be included in the analysis. Unadjusted models and models adjusted for daily average temperature and humidity with same lag periods were evaluated. To examine if associations between daily PM_2.5_ and hospitalizations differed by annual average PM_2.5_, we stratified at the median cut point (9.40 μg/m^3^) of annual average PM_2.5_ concentration for individuals and conducted analyses in both higher and lower long-term exposure strata. By stratifying a population within a case-crossover study design into two groups based on annual exposure to PM_2.5_ we created two distinct populations that cannot be compared statistically, as a result we examined departure from the unstratified estimate as evidence of potential modification.

### Sensitivity analyses

As a sensitivity analysis we examined a stricter definition of COPD. A subset of the population used for this analysis was created by identifying those who had at least 2 hospital visits for COPD during the timeframe of interest and subsequent visits after their two COPD-related visits were examined. We also examined potential differences by number of hospital visits by an individual. We performed analyses stratified by those who had two or more hospitalizations for any cause after their COPD diagnosis wash period and those who only had one hospitalization after their COPD diagnosis to evaluate the potential for severity of COPD to impact results. Finally, we conducted a sensitivity analysis excluding readmissions that occurred on the same day as a previous discharge, as these visits may not be separate from the prior hospitalization.

## Results

### Descriptive information on this cohort and hospital admissions

There were 520 patients with COPD identified from the electronic health records with any hospitalizations at least thirty days after their first COPD visit. Among those, 457 (88%) had at least one respiratory visit and 464 (89%) had at least one cardiovascular-related visit. There were 1,849 hospital visits for any cause, 1,365 for respiratory-related causes and 1,570 for cardiovascular-related causes among this cohort of 520 people with COPD over 12 years. Of the included individuals, 71% identified as white, 26% as Black, and 3% as other race (Table 2). More than half of the cohort (56%) identified as female, and the average age at first visit for patients in this cohort was 65 years. Examining differences across strata of higher and lower annual average PM_2.5_ concentrations, the population in areas with higher annual PM_2.5_ concentrations had a higher proportion of Black patients than the strata with lower annual PM_2.5_ exposure. A larger proportion of those in the higher annual PM_2.5_ concentration strata were identified as deceased (57.25%) in the electronic health records than in the lower annual PM_2.5_ concentration strata (40.86%).

**Table 2:** Descriptive information on cohort of people with at least one hospitalization after COPD hospitalization (*N* = 520)

Characteristic	Total N (%)	Higher^a^ annual PM_2.5_ N (%)	Lower^a^ annual PM_2.5_ N (%)
**N**	*N* = 520	263	257
**Sex**			
Male	229 (44.04)	121 (46.01)	108 (42.02)
Female	290 (55.77)	141 (53.61)	149 (57.98)
**Race**			
Black	135 (25.96)	78 (29.66)	57 (22.18)
White	371 (71.35)	176 (66.92)	195 (75.88)
Other	14 (2.69)	9 (3.42)	5 (1.94)
Deceased by end of follow up	255 (49.13)	150 (57.25)	105 (40.86)
**Average age at first visit**	65 years	64 years	66 years

^a^above and below median concentration

### Exposure and covariate distribution

Among all-cause hospital visits, 0–2 day daily average PM_2.5_ concentrations ranged from 1.00–37.69 μg/m^3^ with a median of 8.77 for event periods and from 0.90–57.17 μg/m^3^ with a median value of 8.83 for control periods (Table 3); these were similar for 0–3 day daily average concentrations. Among those with an address in areas with higher annual PM_2.5_ concentrations (greater than or equal to 9.40 μg/m^3^) 0–2 day daily average PM_2.5_ concentrations ranged from 2.65–34.04 μg/m^3^ with a median of 9.62 for event periods and from 2.06–38.98 with a median of 9.42 for control periods. Comparatively, for patients in areas with lower annual PM_2.5_ concentrations, 0–3 day daily average PM_2.5_ concentrations ranged from 1.00–37.69 μg/m^3^ with a median of 6.32 for event periods and from 0.90–57.17 μg/m^3^ with a median of 8.25 for control periods. Distributions of daily average temperature and humidity did not vary substantially by subgroup; the distribution of each is shown in Tables 3 and 4.

**Table 3:** 0–2 day lag daily average air pollution and meteorology distribution of case days and control days for all cause hospitalizations

PM_2.5_ (μg/m^3^)						
Strata	Type	Min	25th pctl	Median	75th pctl	Max
Overall Population	Cases	1.00	6.70	8.77	11.33	37.69
	Controls	0.90	6.73	8.83	11.33	57.17
Higher Annual PM_2.5_	Cases	2.65	7.15	9.62	12.81	34.04
	Controls	2.06	7.11	9.42	12.37	38.98
Lower Annual PM_2.5_	Cases	1.00	6.32	8.13	10.15	37.69
	Controls	0.90	6.43	8.25	10.47	57.17

**Table 4:** 0–2 day lag daily average air pollution and meteorology distribution of case days and control days for all cause hospitalizations

		Temperature (^o^Farenheit)					Humidity (%)				
Strata	Type	Min	25th pctl	Median	75th pctl	Max	Min	25th pctl	Median	75th pctl	Max
Overall Population	Cases	15.77	44.3	58.73	71.57	82.8	42.1	64.87	71.67	78.3	95.07
	Controls	12.8	45	58.97	71.77	82.77	36.17	64.67	71.67	78.5	94.83
Higher Annual PM_2.5_	Cases	16.03	44.3	58.05	70.77	82.63	46.23	63.37	69.93	77.47	94.27
	Controls	12.8	44.8	57.72	70.63	82.77	38.37	63.7	70.45	77.4	94.83
Lower Annual PM_2.5_	Cases	15.77	43.93	59.73	72.43	82.8	42.1	66.53	72.83	79.43	95.07
	Controls	13.53	45.13	59.7	72.7	82.5	36.17	65.66	72.8	79.4	94.73

*Min* minimum, *pctl* percentile, *Max* maximum

### Associations between daily average PM_2.5_ concentrations and hospitalizations

In unadjusted models, observed associations between short-term PM_2.5_ exposure and hospitalizations for all-cause, cardiovascular-related and respiratory-related hospitalizations were generally null, with ORs per a 5 μg/m^3^ increase in PM_2.5_ of 0.999 (0.927, 1.075), 0.973 (0.901, 1.049) and 0.970 (0.889, 1.060), respectively (Table 5). In models adjusted for daily average temperature and humidity, all-cause hospitalization associations remained generally null but point estimates were shifted slightly upward, while cardiovascular and respiratory hospitalization associations were relatively unchanged with ORs per 5 μg/m^3^ increase in PM_2.5_ of 1.007 (0.936, 1.086) for all-cause hospitalizations with a 2-day lag, 1.003 (0.927, 1.086) for all-cause hospitalizations with a 0–3 day lag, 0.976 (0.900, 1.058) for cardiovascular-related hospitalizations with 0–2 day lag and 0.970 (0.884, 1.065) for respiratory-related hospitalizations with 0–3 day lag.

**Table 5:** Associations (OR (95% CI)) between a 5 µg/m3 increase in daily average PM_2.5_ and hospitalizations, unadjusted and adjusting for average temperature and humidity (*N* = 520)

Event type and exposure timing	Model	Overall	Among higher^a^ annual PM_2.5_	Among lower^a^ annual PM_2.5_
**Any Hospitalization (*N* = 1,849)**				
0–2 day PM_2.5_	Unadjusted	1.003 (0.936, 1.074)	1.060 (0.969, 1.159)	0.919 (0.820, 1.031)
	Adjusted	1.007 (0.936, 1.083)	1.068 (0.968, 1.178)	0.923 (0.820, 1.038)
0–3 day PM_2.5_	Unadjusted	0.999 (0.927, 1.075)	1.060 (0.961, 1.165)	0.910 (0.803, 1.030)
	Adjusted	1.003 (0.927, 1.086)	1.066 (0.958, 1.185)	0.914 (0.804, 1.039)
**Cardiovascular Hospitalizations (*N* = 1,568)**				
0–2 day PM_2.5_	Unadjusted	0.972 (0.901, 1.049)	1.034 (0.935, 1.144)	0.887 (0.784, 1.005)
	Adjusted	0.976 (0.900, 1.058)	1.044 (0.935, 1.167)	0.887 (0.781, 1.009)
**Respiratory Hospitalizations (*N* = 1,379)**				
0–3 day PM_2.5_	Unadjusted	0.972 (0.890, 1.061)	1.030 (0.919, 1.154)	0.890 (0.770, 1.028)
	Adjusted	0.971 (0.885, 1.066)	1.034 (0.911, 1.175)	0.889 (0.766, 1.031)

^a^above and below median concentration

### Associations between daily average PM_2.5_ concentrations and hospitalizations stratified by annual PM_2.5_ concentrations

**Fig. 1:**
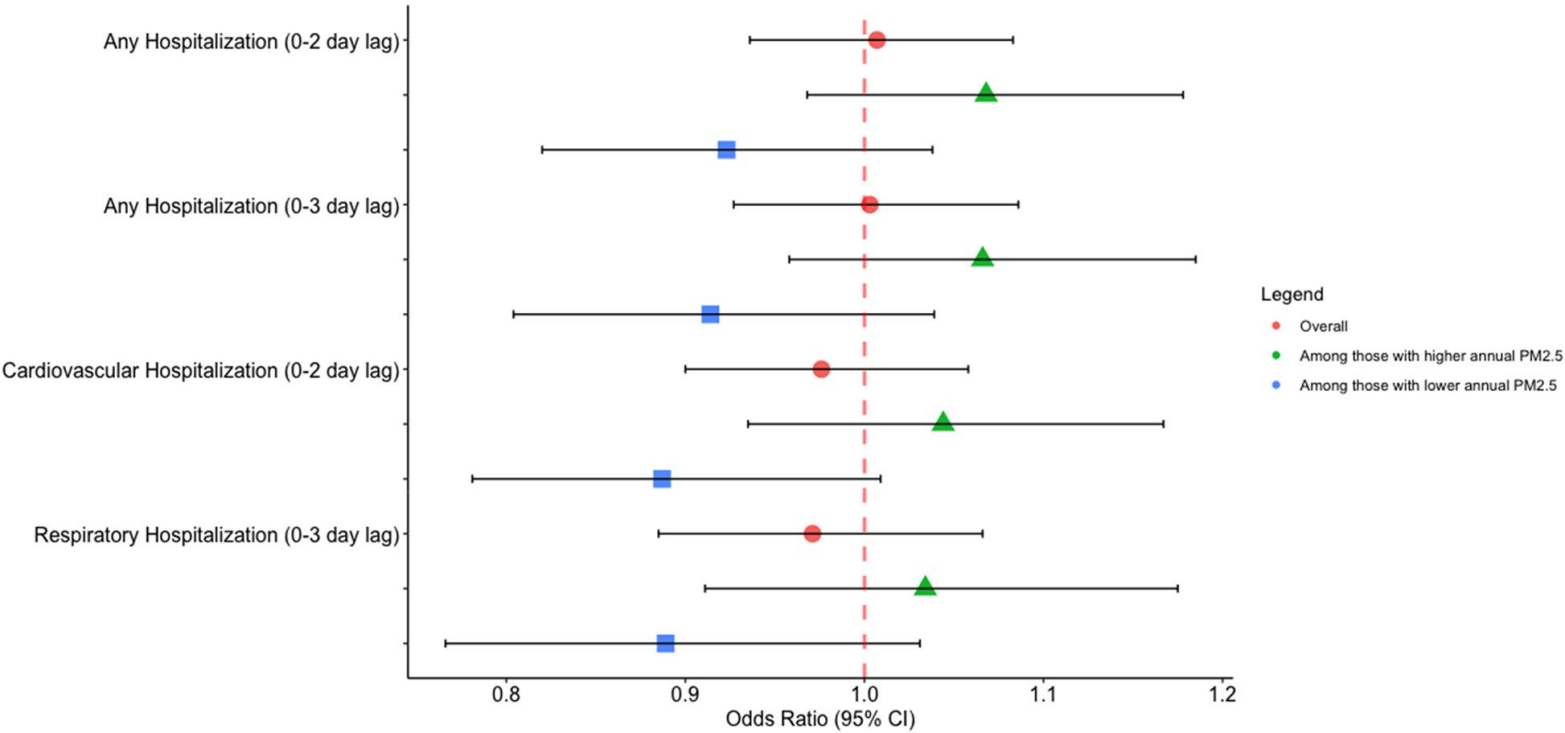
Adjusted Odds Ratio and 95% Confidence Intervals for a 5 µg/m3 increase in short-term PM_2.5_ and hospitalizations among individuals with Chronic Obstructive Pulmonary Disorder, overall and stratified above and below the median of long-term (annual average) PM_2.5_

Differences were observed in the associations between daily average PM_2.5_ concentrations and hospitalizations stratified by annual average PM_2.5_ concentrations with associations larger in magnitude observed among those who live in areas with higher annual PM_2.5_ concentrations. In unadjusted analyses among patients who lived in areas with higher annual average PM_2.5_ concentrations, the OR for hospitalization associated with a 5 μg/m^3^ increase in PM_2.5_ exposure was 1.060 (0.969, 1.159) and 1.058 (0.961, 1.165) for 2-day and 3-day lag exposures, respectively. Alternatively, for those living in areas with lower annual PM_2.5_ concentrations, the OR estimating the odds of hospitalization are 0.919 (0.820, 1.031) and 0.910 (0.803, 1.030) for 2- and 3-day lag exposures, respectively. Similar trends were observed for both cardiovascular and respiratory-related outcomes (Table 5). When adjusting for temperature and humidity, similar trends were observed, with stratified ORs showing a greater trend in association with PM_2.5_ concentrations in places with higher annual PM_2.5_ (Table 5, Fig. 1).

### Sensitivity analyses

Among the original population of 520 people with COPD, 461 people had two or more visits where COPD was their primary diagnosis; therefore, a subset of these 461 confirmed COPD cases was created. The distribution of demographic characteristics of those with two or more COPD visits did not differ from the distribution among our overall population (Table S1). Unadjusted and adjusted associations among this subset of 461 people did not differ from the associations examined in our overall populations (Tables S2 and S3). Among all COPD patients with hospitalizations, 308 patients had at least two hospitalizations following their 30 day wash period and 251 and 273 had at least two visits for respiratory and cardiovascular-related outcomes, respectively. Crude and adjusted models stratified by those with multiple hospitalizations did not differ by much but were slightly higher than the associations for those with only one hospitalization (Tables S4 and S5). Among those who had only one hospitalization 43% died during the study period compared with 54% of those who had multiple visits. Finally, 42 hospitalizations had an admission date that occurred on the discharge date of a previous visit. When these rehospitalizations were excluded, the crude and adjusted associations between air pollution and hospitalizations did not differ from primary analyses (Tables S6 and S7).

## Discussion

Overall, in this cohort of 520 patients with COPD living in North Carolina from 2004–2016, short-term PM_2.5_ exposures were not strongly associated with hospitalization for respiratory-related, cardiovascular-related, or all-cause hospitalizations when not considering the potential implications of long-term PM_2.5_ exposures on health. However, there was some evidence that residence in areas with higher or lower long-term PM_2.5_ concentrations could modify the associations between short-term exposure to PM_2.5_ and hospitalization with those residing in areas with higher annual PM_2.5_ concentrations showing associations larger in magnitude between daily PM_2.5_ concentrations and hospitalizations.

In general, the associations between short-term PM_2.5_ exposure and hospitalizations for all outcomes examined in our study were smaller in magnitude than the associations reported in other studies of the general population [17, 20] and among people with COPD. However, the ambient PM_2.5_ concentrations were higher in previous studies [16, 18]. This is a much sicker population than the general population so they may be on medications to stabilize their symptoms which may result in fewer hospitalizations due to increases in air pollution exposure compared with other populations. Additionally, due to the nature of this cohort of patients with COPD they may be indoors more and are unlikely to have outdoor jobs and therefore may experience a lower burden of increases in air pollution than those who are younger and generally healthier. Furthermore, there is a low variation of air pollution exposure within this population situated primarily within the same region of North Carolina. It is also possible that there is limited evidence of an association between short-term PM_2.5_ exposure and hospitalizations among this small sample size of people living with COPD and using the University of North Carolina (UNC) Healthcare system because they are seeking care for their chronic condition and may be practicing protective behaviors like staying inside on days with poor air quality.

The potential for long-term PM_2.5_ exposure to modify responses to short-term air pollution exposures has not been extensively examined. One study found that the association between short-term PM_2.5_ exposure and mortality remains elevated even in areas with low annual PM_2.5_concentrations [47]. Further understanding of how these exposures might interact, or indicate how individuals react within their own environments to alter direct exposures, is also not well understood. Speculatively, individuals living in areas with generally higher long-term air pollution may be less aware of days with peaks in air pollutant concentrations and would not necessarily alter behaviors to avoid exposures. Or it might be that those who are exposued to higher levels of air pollution regularly experience more inflammation chronically and are therefore more likely to be hospitalized when there are short term increases in air pollution exposure. Additionally, it is possible that those who are more socioeconomically disadvantaged live in areas with higher levels of annual pollution and may have less access to preventative care. Again, given the current sparse state of the literature on this topic, there is much to speculate about, but little evidence to inform our scientific understanding of the relationship between short- and long-term air pollution exposures.

Our study relies on the assumption that the concentration–response relationship between short-term PM_2.5_ exposure and hospitalizations among COPD patients is linear across the entire distribution of PM_2.5_ concentrations observed in our analyses. Specifically, we are comparing a standard unit increase in PM_2.5_ concentration (i.e., 5 μg/m^3^), and thus we assume that the risk in a 5 μg/m^3^ increase is the same in areas with lower annual PM_2.5_ concentrations (e.g., an increase from 6 to 11 μg/m^3^) as it is in areas with higher annual PM_2.5_ concentrations (e.g., an increase from 8 to 13 μg/m^3^). There is evidence to support this assumption; a limited number of studies evaluating the shape of the concentration–response relationship for short-term PM_2.5_ exposure and health outcomes generally report linear relationships [48]. However, if the concentration–response relationship were supra-linear (i.e., higher risks at lower concentrations), or sub-linear (i.e., lower risks at lower concentrations), the generalizability of the results of this study, where the greatest density of PM_2.5_ concentrations is predominately at the lower end of the distribution, to studies conducted in locations where the distribution of PM_2.5_ concentrations includes much higher concentrations would be limited.

While not examined in this study co-pollutant confounding may also be a potential concern. However, to date studies have provided limited evidence of confounding by other co-occurring pollutants. The most recent full assessment of potential confounding of associations between PM_2.5_ exposures and health outcomes concluded that associations remained relatively unchanged when adjusting for other criteria air pollutants in the examination of short-term exposures, with more limited but corroborating evidence when examining long-term exposures [48].

In this study we utilized a small population of people with COPD within the UNC Healthcare system which could affect precision and power of the effect estimates and be a limitation in our analyses. We hope in the future to expand this work to larger populations and more complex methods for examining modification and interaction. By focusing on hospitalization occurrence as the unit of observation and using a case-crossover design, we did not need to adjust for individual- and community-level confounders that do not vary across time. Our study may be subject to some selection bias by only including those who use UNC healthcare for COPD care as these people may be overall sicker and stay inside more often than healthy individuals; they therefore may not be exposed to as much of the increases in PM_2.5_ as others who were not captured in this population. Specifically, our study may suffer from index event bias due to the risk of having COPD and survival to inclusion in this cohort being affected by the exposure PM_2.5_which may bias our results closer to the null [49]. In addition there is the possibility of competing risk from out-of-hospital death, which could potentially lead to a lower observed association, as those who are most susceptible to the impacts of air pollution exposures would be removed from the analysis dataset. Despite this, the use of EHR data is a strength as we were able to capture detailed and accurate health information that can be linked to highly resolved exposure data using residential address. In addition to being highly temporally and spatially resolved, the ensemble model used to estimate PM_2.5_ concentrations has complete coverage for the study catchment area and time period.

## Conclusions

The findings from this study are important for answering public health questions about who is at highest risk of poor health outcomes from exposure to spikes in short-term air pollution exposure. Additional research on how exposure to short-term PM_2.5_ concentration interacts with long-term exposure to PM_2.5_ concentrations, especially among people with chronic conditions like COPD, will be informative. Public health recommendations currently advise people with COPD to seek protection from air pollution on days with higher concentrations of air pollution, specifically PM_2.5_, and further research could inform if there is a need to strengthen these recommendations for people living in areas with higher annual PM_2.5_concentrations. These findings demonstrate the need to examine the joint and interacting effects of long- and short-term air pollution exposures, along with additional conditions and exposures that may increase the risk of people experiencing an air pollution-related health effect.

### Supplementary Information

**Additional file 1.** 

## Data Availability

The datasets generated and analyzed during the current study are not publicly available due to the presence of identifiable information. Code files can be requested from corresponding author (Rappazzo.kristen@epa.gov).

## Abbreviations

COPD: Chronic Obstructive Pulmonary Disease
CVD: Cardiovascular Disease
EPA: Environmental Protection Agency
EHR: Electronic Health Records
NLDAS: North American Land Data Assimilation System Phase 2
OR: Odds Ratio
PM: Particulate Matter
