# Supplementary material for: Effect measure modification of the association between short-term exposures to PM2.5 and hospitalizations by long-term PM2.5 exposure among a cohort of people with Chronic Obstructive Pulmonary Disease (COPD) in North Carolina, 2002–2015
Source: Environ Health. 2023 Jun 29;22:49. doi: 10.1186/s12940-023-00999-4 (PMC10308617; doi:10.1186/s12940-023-00999-4)
Supplement: Supplementary file 1 — Additional file 1. [file 12940_2023_999_MOESM1_ESM.docx]

**Supplemental Materials**

**Interaction between long-term and short-term residential exposure to PM2.5 and the association with hospitalizations among a cohort of people with Chronic Obstructive Pulmonary Disease (COPD) in North Carolina, 2002-2015**

Kristen N. Cowan, Lauren H. Wyatt, Thomas J. Luben, Jason D Sacks, Cavin Ward-Caviness, Kristen M. Rappazzo

Table S1: Descriptive information on cohort of people with at least one hospitalization after two COPD hospitalizations (N=461)

| **Characteristic** | **Total N (%)** | **Higher annual air pollution N (%)** | **Lower annual air pollution N (%)** |
| --- | --- | --- | --- |
| **N** | N=461 | 242 | 219 |
| **Sex** |  |  |  |
| Male | 205 (44.57) | 107 (46.12) | 98 (42.98) |
| Female | 255 (55.43) | 125 (53.88) | 130 (57.02) |
| **Race** |  |  |  |
| Black | 111 (24.13) | 65 (28.02) | 46 (20.18) |
| White | 338 (73.48) | 160 (69.97) | 178 (78.07) |
| Other | 11 (2.39) | 7 (3.02) | 4 (1.76) |
| **Average age at first visit** | 65 years | 65 years | 66 years |

Table S2: Crude Associations (OR (95% CI)) between daily PM_2.5_ and Hospitalizations among subset of the population that had at least two COPD diagnoses (N=461)

| Event type and exposure timing | Overall | Among higher annual PM_2.5_ | Among lower annual PM_2.5_ |
| --- | --- | --- | --- |
| **Any Hospitalization** |  |  |  |
| 0-2 day PM_2.5_ | 0.988 (0.919, 1.062) | 1.045 (0.952, 1.148) | 0.902 (0.799, 1.019) |
| 0-3 day PM_2.5_ | 0.987  (0.913, 1.066) | 1.044  (0.944, 1.154) | 0.899 (0.789, 1.025) |
| **Cardiovascular Hospitalizations** |  |  |  |
| 0-2 day PM_2.5_ | 0.958 (0.885, 1.038) | 1.015 (0.913, 1.127) | 0.878 (0.770, 1.001) |
| **Respiratory Hospitalizations** |  |  |  |
| 0-3 day PM_2.5_ | 0.965 (0.883, 1.056) | 1.023 (0.911, 1.150) | 0.884 (0.762, 1.025) |

Table S3: Associations (OR (95% CI)) between daily PM_2.5_ and Hospitalizations adjusting for daily average temperature and humidity among subset of the population that had at least two COPD diagnoses (N=461)

| Event type and exposure timing | Overall | Among higher annual PM_2.5_ | Among lower annual PM_2.5_ |
| --- | --- | --- | --- |
| **Any Hospitalization** |  |  |  |
| 0-2 day PM_2.5_ | 0.990 (0.917, 1.069) | 1.049 (0.945, 1.163) | 0.905 (0.799, 1.026) |
| 0-3 day PM_2.5_ | 0.989 (0.910, 1.075) | 1.047 (0.937, 1.171) | 0.902 (0.789, 1.033) |
| **Cardiovascular Hospitalizations** | |  |  |
| 0-2 day PM_2.5_ | 0.960 (0.882, 1.045) | 1.020 (0.908, 1.146) | 0.877 (0.766, 1.003) |
| **Respiratory Hospitalizations** |  |  |  |
| 0-3 day PM_2.5_ | 0.963 (0.875, 1.060) | 1.022 (0.897, 1.165) | 0.882 (0.758, 1.028) |

Table S4: Crude Associations (OR (95% CI)) between daily PM_2.5_ and Hospitalizations stratified by number of visits

| Event type and exposure timing | Overall | Among higher annual PM_2.5_ | Among lower annual PM_2.5_ | | Overall | | Among higher annual PM_2.5_ | Among lower annual PM_2.5_ |
| --- | --- | --- | --- | --- | --- | --- | --- | --- |
|  | Among those with 1 hospitalization (N=212) | | | | among those with >1 hospitalization (N=308) | | | |
| **Any Hospitalization** |  |  | |  | |  |  |  |
| 0-2 day PM_2.5_ | 0.988 (0.919, 1.062) | 1.045 (0.952, 1.148) | 0.902 (0.799, 1.019) | | 1.010 (0.939, 1.085) | | 1.080 (0.985, 1.183) | 0.898 (0.792, 1.018) |
| 0-3 day PM_2.5_ | 0.987  (0.913, 1.066) | 1.044  (0.944, 1.154) | 0.899 (0.789, 1.025) | | 1.002 (0.927, 1.082) | | 1.076 (0.976, 1.188) | 0.881 (0.770, 1.009) |
| **Cardiovascular Hospitalizations** |  |  |  | |  | |  |  |
| 0-2 day PM_2.5_ | 0.958 (0.885, 1.038) | 1.015 (0.913, 1.127) | 0.878 (0.770, 1.001) | | 1.000 (0.923, 1.084) | | 1.064 (0.960, 1.181) | 0.901 (0.787, 1.033) |
| **Respiratory Hospitalizations** |  |  |  | |  | |  |  |
| 0-3 day PM_2.5_ | 0.965 (0.883, 1.056) | 1.023 (0.911, 1.150) | 0.884 (0.762, 1.025) | | 0.977 (0.891, 1.072) | | 1.066 (0.947, 1.199) | 0.844 (0.718, 0.992) |

Table S5: Associations (OR (95% CI)) between daily PM_2.5_ and Hospitalizations adjusting for daily average temperature and humidity stratified by number of visits

| Event type and exposure timing | Overall | Among higher annual PM_2.5_ | Among lower annual PM_2.5_ | | Overall | | Among higher annual PM_2.5_ | Among lower annual PM_2.5_ |
| --- | --- | --- | --- | --- | --- | --- | --- | --- |
|  | Among those with 1 hospitalization (N=212) | | | | among those with >1 hospitalization (N=308) | | | |
| **Any Hospitalization** |  |  | |  | |  |  |  |
| 0-2 day PM_2.5_ | 0.919 (0.729, 1.174) | 0.745 (0.491, 1.130) | 1.031 0.774, 1.373) | | 1.017 (0.942, 1.099) | | 1.096 (0.990, 1.214) | 0.901 (0.792, 1.025) |
| 0-3 day PM_2.5_ | 0.942 (0.721, 1.233) | 0.740 (0.472, 1.157) | 1.085 (0.783, 1.503) | | 1.010 (0.930, 1.097) | | 1.093 (0.980, 1.220) | 0.885 (0.770, 1.018) |
| **Cardiovascular Hospitalizations** |  |  |  | |  | |  |  |
| 0-2 day PM_2.5_ | 0.719 (0.543, 0.953) | 0.664 (0.429, 1.027) | 0.762 (0.532, 1.092) | | 1.011 (0.928, 1.102) | | 1.082 (0.965, 1.214) | 0.910 (0.791, 1.047) |
| **Respiratory Hospitalizations** |  |  |  | |  | |  |  |
| 0-3 day PM_2.5_ | 0.932 (0.711, 1.222) | 0.718 (0.470, 1.098) | 1.125 (0.803, 1.578) | | 0.922 (0.885, 1.080) | | 1.077 (0.942, 1.230) | 0.840 (0.711, 0.992) |

Table S6: Crude Associations (OR (95% CI)) between daily PM_2.5_ and Hospitalizations excluding same day rehospitalizations

| Event type and exposure timing | Overall | Among higher annual PM_2.5_ | Among lower annual PM_2.5_ |
| --- | --- | --- | --- |
| **Any Hospitalization** |  |  |  |
| 0-2 day PM_2.5_ | 1.003 (0.935, 1.075) | 1.052 (0.961, 1.151) | 0.930 (0.828, 1.044) |
| 0-3 day PM_2.5_ | 0.998 (0.926, 1.076) | 1.049 (0.952, 1.156) | 0.919 (0.810, 1.043) |
| **Cardiovascular Hospitalizations** |  |  |  |
| 0-2 day PM_2.5_ | 0.970 (0.898, 1.047) | 1.023 (0.924, 1.133) | 0.894 (0.788, 1.014) |
| **Respiratory Hospitalizations** |  |  |  |
| 0-3 day PM_2.5_ | 0.972 (0.889, 1.062) | 1.021 (0.910, 1.145) | 0.900 (0.777, 1.043) |

Table S7: Associations (OR (95% CI)) between daily PM_2.5_ and Hospitalizations adjusting for daily average temperature and excluding same-day rehospitalizations

| Event type and exposure timing | Overall | Among higher annual PM_2.5_ | Among lower annual PM_2.5_ |
| --- | --- | --- | --- |
| **Any Hospitalization** |  |  |  |
| 0-2 day PM_2.5_ | 1.007 (0.935, 1.084) | 1.060 (0.959, 1.171) | 0.933 (0.828, 1.051) |
| 0-3 day PM_2.5_ | 1.003 (0.925, 1.087) | 1.056 (0.949, 1.177) | 0.923 (0.811, 1.052) |
| **Cardiovascular Hospitalizations** | |  |  |
| 0-2 day PM_2.5_ | 0.973  (0.896, 1.056) | 1.033 (0.923, 1.156) | 0.893 (0.784, 1.016) |
| **Respiratory Hospitalizations** |  |  |  |
| 0-3 day PM_2.5_ | 0.972 (0.884, 1.069) | 1.027 (0.903, 1.168) | 0.898 (0.772, 1.045) |
